# Genome Sequence of a Novel H10N9 Avian Influenza Virus Isolated from Chickens in a Live Poultry Market in Eastern China

**DOI:** 10.1128/genomeA.00386-13

**Published:** 2013-06-27

**Authors:** Chunhu Su, Sujuan Chen, Xing Liu, Jing Xu, Kai Huang, Kaichun Liu, Jun Huang, Daxin Peng, Xiufan Liu

**Affiliations:** Animal Infectious Disease Laboratory, College of Veterinary Medicine, Yangzhou University, Yangzhou, Jiangsu, China; Ministry of Education, Key Lab for Avian Preventive Medicine, Yangzhou University, Yangzhou, Jiangsu, China

## Abstract

An H10N9 avian influenza virus (AIV) strain, A/Chicken/Jiangsu/RD5/2013, was isolated in China. The hemagglutinin (HA) and neuraminidase (NA) genes in this strain originated from H10N1 and H7N9 AIVs, respectively, and the other genes derived from H7N3 AIVs. Sequence analysis implies that the H10N9 AIV may be an NA gene donor for the human H7N9 influenza viruses.

## GENOME ANNOUNCEMENT

A new emerging H7N9 influenza virus subtype has become a significant public health threat (1, 2). It is supposed that its hemagglutinin (HA) and neuraminidase (NA) genes originate from Eurasian avian influenza viruses (AIV), and the other genes are from the H9N2 AIV subtypes (3, 4). However, the source of both HA and NA genes remains unknown. To date, the H7N9 influenza virus has not acquired a consistent ability to transmit from human to human, but H7N9 AIV infections from birds to humans are associated with live poultry markets (5). Recently, H7N9, H10N9, and H11N9 AIV subtypes have been isolated from wild birds (6–9). It is important to investigate whether these AIV subtypes exist in live poultry markets, which may provide an NA gene for the H7N9 influenza virus.

In March 2013, an H10N9 AIV strain, A/Chicken/Jiangsu/RD5/2013 (CKRD5), was isolated from chickens in a live poultry market in Jiangsu, China. The eight genes were amplified by reverse transcription PCR (RT-PCR) using a set of universal primers (10). The PCR products were sequenced by GenScript (Nanjing, China).

The full lengths of the polymerase basic 2 (PB2), PB1, polymerase acidic (PA), HA, nucleoprotein (NP), NA, matrix (M), and nonstructural (NS) genes were 2,341, 2,341, 2,233, 1,765, 1,565, 1,467, 1,027, and 890 nucleotides, respectively. The amino acid sequence of the cleavage site in the HA protein was PEIMQER↓GLF, which was different from PEIMQGR↓GLF in the other H10 subtype AIV. The amino acid residues at the receptor binding site were Q226 and G228 (H3 numbering), an avian-like receptor binding specificity. The amino acids at residues 627 and 701 in the PB2 protein were glutamic acid and aspartic acid, respectively, a characteristic of avian replication preference. There were no deletions in the NA and NS genes. In contrast, there are five amino acid deletions at the NA stalk region in human H7N9 influenza viruses (4).

Sequence analysis showed that the nucleotide sequences of the HA gene of the CKRD5 virus were closely related to those of the strain A/wild bird/Korea/A12/2010 (H10N1) (11) and shared 96.7% nucleotide homology. The nucleotide sequences of the NA gene of the CKRD5 virus were closely related to those of A/wild bird/Korea/A14/2011 (H7N9) and shared 98.1% nucleotide homology. At the same time, the NA gene of the CKRD5 virus shared 98.7% nucleotide homology and 99.1% amino acid homology with those of strain A/Anhui/1/2013 (H7N9). The other six genes (PB2, PB1, PA, NP, M, and NS) of the CKRD5 virus were closely related to those of H7N3 AIVs, e.g., A/duck/Zhejiang/12/2011(H7N3), which were isolated from domestic ducks in live poultry markets in Zhejiang Province, China, in 2011 (12). They shared 98.8%, 98.1%, 97.8%, 98.5%, 99.6%, and 99.2% nucleotide homologies with those six genes of the H7N3 AIVs, respectively.

In conclusion, the CKRD5 virus is a reassortant with the HA and NA genes from wild bird origin H10N1 and H7N9 AIVs, respectively, and the other six genes from H7N3 AIVs. The CKRD5 virus may be an NA gene donor for the emerging H7N9 influenza viruses.

### Nucleotide sequence accession numbers.

The complete genome sequence of A/Chicken/Jiangsu/RD5/2013 was deposited in GenBank under the accession no. KF006411 to KF006418.
